# Extremely durable biofouling-resistant metallic surfaces based on electrodeposited nanoporous tungstite films on steel

**DOI:** 10.1038/ncomms9649

**Published:** 2015-10-20

**Authors:** Alexander B. Tesler, Philseok Kim, Stefan Kolle, Caitlin Howell, Onye Ahanotu, Joanna Aizenberg

**Affiliations:** 1 John A. Paulson School of Engineering and Applied Sciences, Harvard University, Cambridge, Massachusetts 02138, USA; 2 Wyss Institute for Biologically Inspired Engineering, Harvard University, Cambridge, Massachusetts 02138, USA; 3 Kavli Institute for Bionano Science and Technology, Harvard University, Cambridge, Massachusetts 02138, USA

## Abstract

Formation of unwanted deposits on steels during their interaction with liquids is an inherent problem that often leads to corrosion, biofouling and results in reduction in durability and function. Here we report a new route to form anti-fouling steel surfaces by electrodeposition of nanoporous tungsten oxide (TO) films. TO-modified steels are as mechanically durable as bare steel and highly tolerant to compressive and tensile stresses due to chemical bonding to the substrate and island-like morphology. When inherently superhydrophilic TO coatings are converted to superhydrophobic, they remain non-wetting even after impingement with yttria-stabilized-zirconia particles, or exposure to ultraviolet light and extreme temperatures. Upon lubrication, these surfaces display omniphobicity against highly contaminating media retaining hitherto unseen mechanical durability. To illustrate the applicability of such a durable coating in biofouling conditions, we modified naval construction steels and surgical instruments and demonstrated significantly reduced marine algal film adhesion, *Escherichia coli* attachment and blood staining.

## Abstract (web summary)

Steel is employed ubiquitously throughout human society thanks to its outstanding mechanical properties, however over time degradation can lead to a loss in performance. Here, the authors show that creating liquid-infused surfaces via electrodeposition of a tungstite film endows steel with a durable coating which inhibits corrosion and fouling.

## Graphical abstract

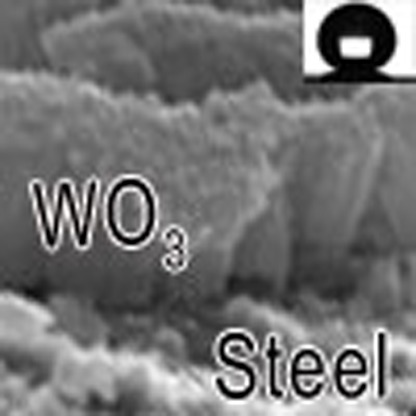

Steels are used almost everywhere from biomedical devices and surgical instruments, household and industrial equipment, to military, transport and architecture. This is due to their superior mechanical properties—strength, ductility, elasticity and high corrosion resistance. Various steel alloys have been engineered to meet different application requirements allowing their incorporation into infrastructure in nearly all environments. For example, corrosion-resistant austenitic steels are used in cutlery, medical implants, chemical industry, and construction and aquatic environment, to name a few^1^,^2^; ferritic steels combine good corrosion resistance with excellent formability and ductility and are mostly used for indoor applications; surgical-grade steels are used in biomedical applications^3^; ultra-light steels were developed to reduce the overall weight of vehicles^4^; high-yield steels are used in military and naval applications^5^,^6^; and duplex alloys (a mixture of austenite and ferrite) were designed for high strength, pitting corrosion resistance applications, such as heat exchangers, pressure vessels and chemical processing industries^7^.

Interaction of steel with various liquids, often aggressive and highly fouling, is common in most applications, and it generally leads to significant reduction in function, corrosion and contamination. In particular, a variety of industries worldwide are plagued by biofouling^8^. For instance, marine biofouling introduces surface roughness that causes increased frictional resistance and fuel consumption and decreased top speed and range of the ships^9^. To prevent it, anti-fouling coatings have been widely applied; however, most successful coatings incorporate environmentally toxic biocides that impact non-target species^9^. The costs of microbiologically influenced corrosion of industrial water systems—a phenomenon whereby steel surfaces in contact with water or humid atmosphere are colonized by microorganisms^10^—account for 2–3% of the GDP in developed countries^11^. Finally, surgical site infection—the most common infection acquired during hospitalization^12^,^13^—is caused by microorganisms derived either from the patient skin or from surgical instruments that contaminate a traumatic wound. These postoperative complications dramatically increase hospital length of stay, costs and mortality^12^.

There are numerous approaches in the design of anti-biofouling materials: coating with intrinsically or artificially loaded antimicrobial materials, antibiotic or cationic polymer grafting, micro-nano structuring and photoactivation of surfaces^14^,^15^. The majority of these approaches have substantial limitations, such as mechanical weakness, poor adhesion to substrate, non-uniform coating, leaching of toxic chemicals, lack of long-term stability and biocompatibility. Hence, a simple, non-hazardous and inexpensive method to prepare durable, non-wetting steel surfaces with uncompromised mechanical properties, which repel a variety of contaminants, has immediate relevance in applications that suffer from the above-mentioned issues^16^.

In recent decades, superhydrophobic surfaces (SHS)^17^, which imitate the lotus architecture where water droplets roll off the nanoscopically rough leaf surface^18^, emerged as a potential solution to creating non-fouling materials. Many techniques have been reported to fabricate non-wetting SHS using large variety of materials^18^,^19^,^20^,^21^. However, the ‘Achilles heel' of SHS is poor mechanical and pressure stability of the hierarchical micro/nano surface topography that is essential for obtaining very high contact angles (CAs)^18^. Despite the technological importance of SHS, it is noteworthy that neither natural SHS nor their artificial analogues have good mechanical stability^17^,^22^, and therefore cannot be effectively used in demanding, abrasion-heavy applications, especially to modify metal surfaces. Even more challenging is to create SHS that resist wetting by low-surface-tension liquids and biological fluids or minimize organisms′ attachment.

Recently, slippery liquid-infused porous surfaces (SLIPS) were introduced as an alternative approach to anti-fouling materials^23^. SLIPS function under high-pressure conditions^23^, self-heal imperfections^23^, provide optical transparency^24^, reduce ice nucleation^25^ and are ultra-repellent to complex fluids such as crude oil and brine^23^,^24^, as well as repel highly contaminating biological materials such as blood or biofilms^23^,^24^,^26^. These surfaces can be constructed from a broad range of inexpensive materials; however, approaches described so far do not result in materials that can be applied to steel without significant reduction in durability and are not therefore, suitable for demanding applications. Hence, of particular importance is to extend the SLIPS technology to mechanically robust systems that will match or even improve the mechanical performance of metals and alloys.

To face the durability challenge, transition^27^ and rare earth^28^ metal oxides are emerging as important materials due to their versatile chemical, physical and mechanical properties^29^. Metal oxide films are conventionally grown by vapour deposition under reduced pressure; however, the high cost of equipment, a small coverage area and the difficulty of producing porous films have limited their potential applications. On the other hand, solution-based deposition techniques, such as sol–gel or hydrothermal synthesis, are inexpensive, but limited to particular metal oxides, which commonly have poor adhesion to metallic substrates, and require expensive post-deposition treatments to achieve desired resilience^30^. In particular, tungsten oxides (TO) have been studied extensively in the last few decades due to their unique electro- and photo-chromic properties^31^,^32^, electrocatalytic activity^33^,^34^, excellent resistance to corrosive chemicals under both acidic and basic conditions^35^ and high mechanical durability^29^. Chemical^36^ and electrochemical^32^,^37^ methods have been applied to create tungsten trioxide films for electrochromic devices^32^,^38^,^39^.

In this work, we report exceptionally robust nanoporous TO coatings directly formed on various grade steel surfaces via a facile, room-temperature electrochemical deposition technique. Durability and adhesion of TO films are characterized by nanoindentation, adhesive tape peeling, bending and particle abrasion tests^40^,^41^. The TO coatings are as mechanically durable as the bare steel substrates and maintain their integrity after applying significant abrasive force. We show the use of nanoporous TO films in fabrication of superhydrophobic and liquid-infused surfaces with unique mechanical durability. We demonstrate that our omniphobic-slippery TO coatings are able to retain their morphology and wetting characteristics under high-friction conditions and, at the same time, to completely repel blood and significantly reduce attachment of *Escherichia coli* to medical-grade steel devices. Furthermore, when this coating is constructed on an austenitic-grade stainless steel, a commonly used steel in marine environment^42^, the omniphobic-slippery TO films almost completely repel algae fouling without showing any sign of morphology degradation or environmental toxicity.

## Results

### Structural and chemical characterization of tungstite films

**Figure 1:**
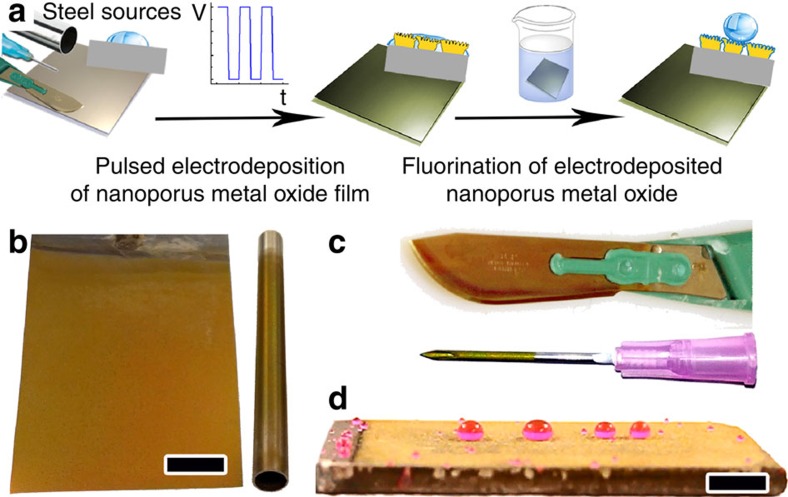
Electrodeposited TO films on a variety of surface geometries and steel grades. (**a**) Schematic representation of the preparation procedure based on electrodeposition of a rough TO film on steel. Insets represent intrinsically hydrophilic steel substrates (water CA ∼70°) that become superhydrophilic (water CA 0°) after electrochemical deposition of a rough TO film, and transform to superhydrophobic wetting state (water CA >170°) after chemical functionalization. (**b**–**d**) TO films were deposited on several working electrode geometries made from various grades of steel: (**b**) an AISI 304 grade stainless-steel sheet and a pipe with 0.5 cm inner diameter, (**c**) a surgical-grade steel scalpel blade and syringe needle coated with TO on both internal and external surfaces, and (**d**) a naval construction HY-100 grade steel plate. Scale bar, 1 cm.

A schematic diagram of the fabrication procedure of SHS based on electrochemical deposition of TO films is shown in Fig. 1a. Rough and porous TO nano-textured films were created through square-waveform, pulsed, cathodic electrodeposition using naturally aerated aqueous sodium tungstate solution at pH>10. This procedure was successfully used to deposit TO films on a wide range of steel alloys and cathode geometries: austenitic-grade stainless-steel AISI 304 and 316 (Fig. 1b), ‘non-hardenable' ferritic AISI 430, surgical (Fig. 1c) and naval construction HY-100 grade steels (Fig. 1d). Application of this approach to large-scale objects may require additional technological improvements, for example, the use of a pulsed DC rectifier in a standard two-electrode electrochemical cell.

**Figure 2:**
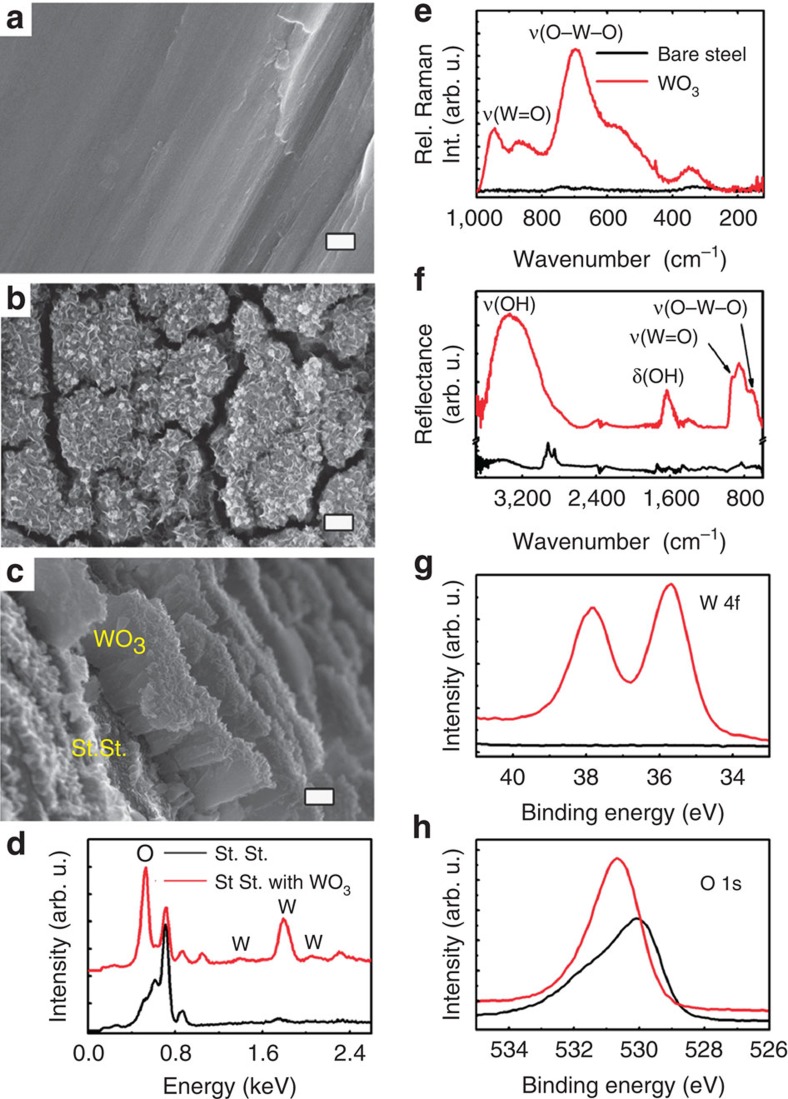
Morphology and elemental composition of electrodeposited TO films on austenitic-grade steel. (**a**) SEM image of a bare AISI 304 stainless-steel surface, (**b**,**c**) SEM images of TO films deposited by square wave-pulsed deposition on stainless steel revealing the deposited film thickness and hierarchically structured island morphology: (**b**) top view, (**c**) tilted view (70°). Scale bar, 200 nm. (**d**) Energy-dispersive X-ray analysis spectra of bare stainless steel and after deposition of TO film. (**e**–**h**) Raman (**e**), FTIR (**f**) and high-resolution XPS spectra of W 4f (**g**) and O 1s (**h**) regions of as-deposited TO films on stainless steel (red lines) compared with the corresponding spectra from the bare stainless-steel substrate (black lines). The stretching mode of the terminal W=O bond (947 cm^−1^) is clearly observed in both Raman and the FTIR spectra characteristic of hydrated TO (**e**,**f**). The Raman spectrum exhibits a single broadband centred at 695 cm^−1^ associated with the O–W–O stretching modes of the bridging oxygen atoms and a band at 370 cm^−1^ is assigned to the W–OH_2_ translational motion (**e**). The OH stretching and H–O–H bending vibrations of the water molecules are clearly identified in the FTIR spectrum around 3,340 and 1,620 cm^−1^, respectively (**f**). The XPS spectra (**g**) consist of a single doublet at binding energies 35.7 eV for the W 4f_7/2_ and 37.8 eV for the W 4f_5/2_ corresponding to the W(VI) oxidation state (see also Supplementary Fig. 3A). The O 1s broad peak of bare stainless steel (**h**) is associated with the O^2−^ (∼530.2 eV) and the OH^−^ (∼531.4 eV) from the native oxides and hydroxides formed on stainless-steel surface. The O 1s spectrum of deposited films shows slightly asymmetric peak centred at 530.9 eV (H) that has been deconvoluted into three components: 530.8 eV (69.4%), 531.8 eV (29.0%) and 532.7 eV (1.6%) corresponding to the O^2−^, OH^−^ and H_2_O, respectively, in the hydrated TO (see Supplementary Fig. 3B).

Formation of cathodically pulsed-deposited TO films can be visually observed by appearance of golden-yellow lustre that alters to yellow-green and finally to opaque yellow-brown with deposition time due to an increase in film thickness and the development of roughness. The morphology of the TO films yielding these colours, comprises a hierarchically structured microscale islands covered with nanoscale flakes with overall thickness of ∼600 nm on flat stainless-steel surface (Fig. 2a–c). Energy-dispersive X-ray analysis confirms that the rough deposited films are composed of TO (Fig. 2d and Supplementary Fig. 1). In contrast, deposition of TO films using continuous mode with similar deposition conditions resulted in formation of thin (10−20 nm) dense films (Supplementary Fig. 2).

Tungsten trioxide and its hydrates have various allotropic modifications, in which W(VI) metal centres hexacoordinated by oxygen atoms are linked in different ways owing to the distortions and easy hydration of WO_3_ by substitution of one oxygen of the octahedra by water molecules and/or by intercalation of water molecules between the sheets of the layered structure^43^. The golden-yellow colour of the deposits indicates the formation of hydrated TO of the type WO_3_·*x*H_2_O (ref. 43). The degree of hydration was characterized by Raman and Fourier transform infrared spectroscopy (FTIR) techniques (Fig. 2e,f)^44^. Appearance of the single broadband at 695 cm^−1^ in the Raman spectrum and OH stretching and H–O–H bending vibrations of the water molecules in the FTIR spectrum suggest that deposited films are monohydrated TO with amorphous structure^43^,^45^. High-resolution XPS spectra of W 4f and O 1s electrons (Fig. 2g,h and Supplementary Fig. 3) show signatures of the W(VI) oxidation state and O^2−^, OH^−^ and H_2_O in the hydrated TO (ref. 46), further confirming that the electrodeposited films consist of WO_3_·H_2_O (tungstite). Similar observations were made when molybdenum oxide, which has strong chemical similarity to TO (refs 43, 47), was electrodeposited from alkali solutions^48^. It has also been shown that WO_3_·H_2_O films adopt flake-like morphology during hydrothermal treatment^49^, and island-like structures are distinctive of pulsed-electrodeposited oxide films^50^,^51^.

Initially hydrophilic TO-modified steel surfaces were rendered superhydrophobic (TO-SHS) by surface modification with perfluoroalkyl-bearing phosphate^52^ and further converted to omniphobic-slippery liquid-infused porous surfaces (TO-SLIPS) by infiltration of fluorinated TO-SHS with perfluoropolyether lubricants.

### Mechanical properties of nanoporous TO films on steel

Metallic tungsten is well known for its exceptional mechanical characteristics such as high hardness and Young's modulus, melting point and resistance against oxidation and corrosion^53^, however the increase in oxygen content during oxidation and development of porosity is expected to affect and generally reduce its mechanical strength^29^. While majority of the research on thin TO films has been focused on probing chromic effects, only few studies have addressed their mechanical stability^29^,^53^.

We have applied various techniques to characterize mechanical properties of hydrated porous TO films^54^. (i) Nanoindentation was used to determine the Young's modulus and hardness. (ii) Scotch tape and bending tests were performed to characterize adhesion of the deposited films to the underlying substrate^55^. (iii) Water and hard particle abrasion tests were performed to demonstrate the resistance of the TO surfaces to massive mechanical damage over a large area^40^.

**Figure 3:**
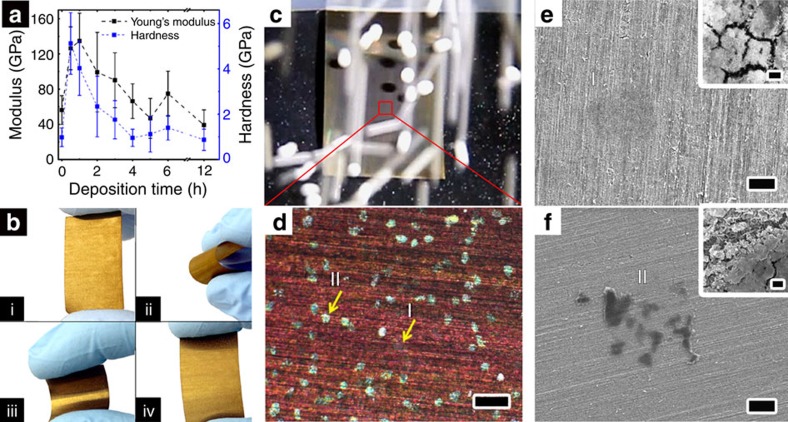
Mechanical resistance of electrodeposited TO films on stainless steel. (**a**) Indentation Young's modulus (black line) and hardness (blue line) of AISI 304 stainless steel before and after electrodeposition of porous TO films as a function of deposition time. (**b**) Bending of 100-μm-thick stainless-steel plate coated with the electrodeposited TO porous film: (i) before bending, (ii and iii) bending in both inward and outward directions and (iv) after 10 times bending in both directions. (**c**) A top view snapshot from a particle abrasion experiment with 2 mm YSZ particles impinging on the samples during test. In each experiment, 35 g of YSZ media was used. Superhydrophobic TO samples were mounted near-normally to the falling particles and the test was repeated 10 times by alternating YSZ particles of 0.8 and 2 mm diameter. (**d**) Bright field reflection optical image of the sample after 10 particle abrasion tests. Labels ‘I' and ‘II' indicate areas abraded by 0.8 and 2 mm particles, respectively. The contrast is mainly due to slight dent reflecting the light differently. The dent on the surface caused by impingement with lighter YSZ particles was hardly observable in optical microscope images. Scale bar, 200 μm. (**e**,**f**) SEM images of the sample shown in **d** displaying surface damage produced by 0.8 (**e**) and 2 mm (**f**) YSZ particles, respectively. Scale bar, 10 μm. Inset images (scale bar, 100 nm) show a higher magnification of the abraded area.

Nanoporous TO coating electrodeposited on AISI 304 substrate was tested by nanoindentation and compared with a bare stainless-steel control (Fig. 3a). During first hour of deposition, TO-coated steel exhibits a significant increase in both Young's modulus and hardness compared with bare steel substrates. Formation of a thin ceramic film strongly adhered to the substrate explains this improvement (Supplementary Fig. 4B,C). With increase in the deposition time, mechanical properties gradually decrease mainly due to the development of film porosity (Supplementary Fig. 4D–H)^56^. Surface roughening is required to increase its hydrophobicity; however, many of the superhydrophobic coatings fail mechanically at this stage^57^. In the current system, no significant decrease in either Young's modulus (*E*=39.2±17.2 GPa versus 56.2±16.4 GPa) or hardness (*H*=0.87±0.47 GPa versus 0.98±0.41 GPa) was observed for the most porous TO-coated films (Fig. 3a). While nanoporous WO_3_·H_2_O shows lower values than previously reported for thick and dense sputtered WO_*x*_ films on steel^29^,^53^, the porous nature of nanocoating, critical for the formation of wetting-resistant surfaces, does not compromise the mechanical properties of the underlying steel.

The adhesion strength of the coating was examined by adhesive tape peel test and by bending of the 100-μm-thick stainless-steel sheets. As-deposited TO films show strong adhesion to the substrates passing Scotch tape tests. In addition, samples were repeatedly bent up to an angle of 180° and examined visually (Fig. 3b) and by high-resolution scanning electron microscopy (HR-SEM) (Supplementary Fig. 5). No sign of delamination or peeling of the coating was observed. After 10 repeated bending cycles, no fracturing, blistering or cracking occurred. Chemical and structural characterization of the coatings discussed above suggests that the excellent adhesion of TO films to steel surfaces is due to the formation of M–O–W bonds during the first few cycles of deposition (where M=Fe, Cr, Ni), while high durability under compressive and tensile stresses arises from the island morphology of the films, with energy dissipation occurring at the 100-nm-wide crevices separating the islands.

Traditional abrasion tests employ water drops or solid particles to impact the surface and characterize the resulting surface damage. Continuous bombardment of the nanostructured TO surfaces with water droplets (volume: 20 μl, droplet rate: 1 droplet per second, impinging height: 5 cm) was performed and no mechanical damage of even the finest nanofeatures was observed. In a typical particle abrasion experiment, superhydrophobic TO surfaces were impinged with yttria-stabilized-zirconia (YSZ) particles with diameters of 0.8 mm (2.1 mg) and 2 mm (28 mg) from a height of 40 cm over ∼2 × 2 cm area, corresponding to an impinging energy densities of 7.8 × 10^−6^ and 1.1 × 10^−4^ J per particle, and an impinging velocity of 10.1 km h^−1^ (Fig. 3c and Supplementary Fig. 6). Such impinging energy densities are three orders of magnitude higher than the highest reported values for the most durable, abrasion-resistant superhydrophobic coatings^40^,^58^. It is noteworthy that the estimated impact energy of a natural ∼50 μm sand particle during real wind-storm conditions (according to National Oceanic and Atmospheric Administration) is just ∼10^−8^ J per particle^58^.

Figure 3d shows an optical microscope image of a sample after 10 particle abrasion tests. Two types of damaged areas were identified: pale, hardly observable spots from 0.8 mm particles (indicated as ‘I') and bright spots from 2 mm particles (indicated as ‘II'). High-resolution SEM images (Fig. 3e,f) highlight the impact produced by 0.8 mm particles and show that it removes some of the fine nanoscale structure from the islands (Fig. 3e, inset), while 2 mm particles push aside parts of the islands, likely exposing unmodified hydrophilic TO fragments (Fig. 3f, inset). Still, from the images it is clear that even after 10 particle abrasion tests, the produced dents are confined to the directly impinged area. The combination of strong adhesion of the TO to the substrate together with the island-like morphology minimizes the propagation of the damage over a larger area.

### Wetting properties of superhydrophobic TO-coated steel

Wetting properties of the TO coatings were quantified by CA, contact angle hysteresis (CAH) and sliding angle measurements using water as a test liquid. Owing to the inherent hydrophilicity of TO and rough topography, the as-deposited films show superhydrophilicity with a CA of ∼0° (ref. 59). After surface modification with perfluoroalkyl-bearing phosphate, the static CA reaches the value of 169°±4°, characteristic of a superhydrophobic surface. However, a droplet of water placed on such surfaces experiences pinning with CAH values of 45°±15°.

Hydrophobicity and the associated repellent performance of non-wetting surfaces can be compromised in several ways: (i) chemical degradation of the modification layer on exposure to ultraviolet light or ozone; (ii) temperature-induced chemical or mechanical degradation; and (iii) mechanical damage. The influence of all these factors on wetting characteristics was studied.

**Figure 4:**
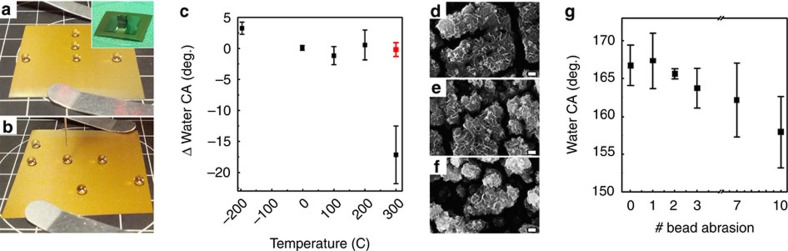
Wetting stability of TO-modified stainless steel exposed to ultraviolet irradiation, and thermal and mechanical abrasion treatments. (**a**,**b**) Images of water droplets (10 μl) on TO-SHS stainless steel before (**a**) and after 1 h ultraviolet irradiation on exposed and masked areas (**b**). Inset image: The sample during ultraviolet exposure protected by stainless-steel mask. The total illumination energy was 2 kJ for exposed sample area (∼7 cm^2^). No changes in surface wettability were observed. (**c**) Changes in water CAs of the electrodeposited TO on stainless steel, measured at room temperature after heating/cooling. (**d**–**f**) Corresponding HR-SEM images of the film morphology: (**d**) As-deposited, (**e**) cooled in liquid nitrogen (−196 °C) and (**f**) annealed for 1 h at 300 °C. Scale bar, 100 nm. The sample annealed at 300 °C was submerged in the modification solution and CA was restored to the original value (red point in **c**). (**g**) Water CAs of the sample before and after first, second, third, seventh and tenth bead abrasion experiments as shown in Fig. 3. On the basis of the geometrical changes owing to the formation of well-defined dents, the increase in liquid–solid contact area of droplets sitting on the damaged surface was calculated and was found to be negligible, emphasizing again that the exposure of the hydrophilic TO fragments was insignificant. These calculations can explain such a minor decrease of the CA values even after significant surface abrasion.

There are several reports showing that rough TO films were transformed from superhydrophobic to superhydrophilic state under ultraviolet illumination either by photoreduction of tungsten atoms^38^ or photodecomposition of the hydrophobic layer assembled on the surface^60^. Illumination of superhydrophobic TO films by a broadband ultraviolet flood lamp for 1 h in air shows no difference in the wetting properties of the illuminated and masked areas (Fig. 4a,b). This result (absence of TO photoactivity) was predicted for the oxygen-saturated TO compounds, as in present films^61^.

In addition, the crystal structure of TO is temperature dependent^62^. Therefore, to determine the morphological and, consequently, wetting stability of the deposited films, samples were either annealed at temperatures up to +300 °C in air, or cooled in liquid N_2_ (−196 °C). No noticeable morphological change of the deposits was observed after low- and high-temperature exposure (Fig. 4c–f). Moreover, the TO samples did not show mechanical degradation, when heated or submerged in liquid nitrogen and consequently bent ∼180° (Supplementary Fig. 7). The CA changes insignificantly in the temperature range between −196 and +200 °C and diminishes slightly above that temperature, likely due to partial pyrolysis of the modification layer (Fig. 4c, black points). When the sample annealed at 300 °C was re-functionalized, the CA was completely restored to the original high value (Fig. 4c, red point).

The most detrimental process that compromises the superhydrophobic character and repellent performance of coatings involves mechanical degradation of the structured solid surface. However, if the damaged area is localized within a tolerable finite dimension, increase in the contact area between repellent liquid and the surface will be minor and wetting characteristics of the surface will be preserved. CA of the superhydrophobic TO samples was measured before and immediately after particle abrasion experiments (Fig. 4g). The surface damage after 10 bead abrasion experiments was ∼25% (calculated as the area of the bright spots from the optical microscopy images). Such surface damage implies the presence of 7–10 damaged spots per 10 μl droplet at the liquid–solid interface. Despite substantial surface damage, the CA values reduced only slightly from 167°±3° to 158°±5°, and the surfaces still remained superhydrophobic (see Supplementary Movie 1).

### Omniphobicity of liquid-infused TO-SLIPS-coated steel

Many advantages are expected when mechanically robust SHS are converted into SLIPS (TO-SLIPS)^23^,^24^. In particular, while SHS show exceptional anti-wetting behaviour against water, they generally fail to repel low-surface-tension liquids^6^, fail at high or low temperature, and show significant contamination in biological media^3^,^7^. When TO-SHS stainless-steel surfaces were infused with the lubricant, the resultant TO-SLIPS samples were resistant to wetting under all the above conditions, simultaneously repelling water, ethanol and silicone oil (Supplementary Movie 2). Owing to high chemical affinity of the fluorinated lubricant to the fluorinated porous solid, the lubricant is stably held in and on the nanostructured surface replacing air trapped inside porous structure and forming a conformal, molecularly smooth liquid overlayer. Liquids that are immiscible with the infused lubricant do not penetrate this layer and experience liquid–liquid interaction at the interface with the lubricant, thus effectively avoiding pinning on the solid and leading to extremely low values of CAH and sliding angles and therefore high droplet mobility (Table 1). Moreover, thick and wide interconnected crevices in the TO coating serve as an additional reservoir to hold the lubricant, and help maintain constant lubricant overlayer on the surface under operation conditions as well as self-heal damaged areas due to high mobility of the liquid lubricant. As a result, as we show in Supplementary Movie 3, even after intense scratching with stainless-steel tweezers, a screw driver and a diamond scriber, the surfaces retain their non-wetting behaviour—the property that has never been achieved in traditional SHS or simple slippery materials^23^,^24^. Importantly, samples that were lubricated and stored for a year under ambient conditions, still exhibited the CAH values well below 5° (Supplementary Fig. 8). The presence of the lubricant overlayer also protects the underlying coating when exposed to highly corrosive etchants (Supplementary Figs 9 and 10).

### Biofouling properties of TO-coated steel substrates

Steel is a commonly used material that often suffers from biological fouling. The process of biofouling is complex, but its beginnings are somewhat similar for biomedical and marine environments, starting with spontaneous non-specific adsorption of organic molecules on the surface followed by colonization by micro-organisms^63^.

Anti-biofouling properties of the TO coatings were investigated using several biological model systems. TO coatings fabricated on surgical-grade stainless-steel scalpel blades and needles (Fig. 1c), were converted to superhydrophobic and, finally, to omniphobic TO-SLIPS using fluorinated Krytox GPL-K103 lubricant or medical-grade perfluorocarbon lubricants, such as perfluorodecalin^64^. The latter is highly biocompatible, and has been previously used in multiple applications including liquid ventilation, ophthalmic surgery and as a blood substitute. In invasive medical procedures, the scalpel blade surface experiences significant friction during incisions made through the skin and subcutaneous tissue. At the same time, these instruments are exposed to pathogens on the patient's skin and to blood, potentially leading to infection^65^. Performance of the omniphobic TO coating was evaluated first by making an incision with the treated blades into viscoelastic silicone rubber slabs of various softness and stickiness (polydimethylsiloxane (PDMS) base/curing agent ratios of 30:1, 20:1 and 10:1) chosen to mimic skin tissues^66^. This was followed by submersion into blood and a concentrated culture of *E. coli*.

**Figure 5:**
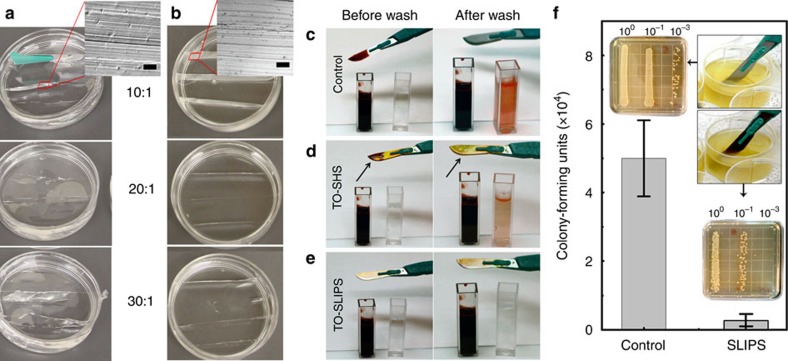
Repellent properties of TO films constructed on surgical-grade stainless-steel scalpels. (**a**,**b**) Incisions produced on 1-cm-thick, sliced PDMS slabs with increased softness and stickiness (base/curing agent ratio, indicated). Insets: optical microscope cross sectional images of silicone slabs sliced by untreated (**a**) and TO-SLIPS scalpel blades (**b**). Scale bars in insets, 50 μm. (**c**–**e**) Scalpels were immersed in a cuvette with blood (left column) and blood staining on the scalpel surface was washed off into a cuvette with DI water (right column): (**c**) Untreated, (**d**) TO-SHS and (**e**) TO-SLIPS. Untreated and TO-SHS scalpels were immersed once; TO-SLIPS scalpel was immersed three times. Colour of the cuvette with DI water indicates and qualitatively correlates with the amount of blood that remains on every sample. (**f**) C.f.u. counts for untreated (control) and TO-SLIPS scalpel blades after dipping in a concentrated culture of *E. coli* followed by a brief rinse in DI water.

When the control, untreated scalpel was used, the softest slab (30:1 PDMS) stuck to the scalpel surface and moved along the direction of cutting creating uneven incision. For the hardest slab (10:1 PDMS), it was almost impossible to cut through because of the friction, occasionally resulting in breaking of the plastic handle of the scalpel blade (Fig. 5a). In all cases, incisions produced by untreated scalpels tended to have non-uniform cuts and fractures due to the stick-slip-like, jerking motion. In contrast, the TO-SLIPS blades produced smooth, nearly identical incisions on all slabs tested (Fig. 5b), despite the presence of the nanoscale rough film on both blade edges.

After incision experiments, untreated, TO-SHS and TO-SLIPS scalpels were submerged in a cuvette with blood (Fig. 5c–e and Supplementary Movie 4). The TO-SHS scalpel lost its repellent property immediately after submersion in blood as evidenced by the adherent blood remaining on its surface and the pinned water film after the wash (Fig. 5d). Qualitatively, the amount of blood was significantly lower on the TO-SHS scalpel than on the control as indicated by water colour after washing. The TO-SLIPS scalpel showed neither blood adhesion nor loss of the slipperiness after multiple submersions in blood (Fig. 5e). The latter result is in good accordance with previously reported blood-repellent properties of SLIPS under static or flow conditions^23^,^24^,^64^, but this important anti-fouling characteristic can now be displayed by a durable material after mechanical contact or damage.

Following these experiments, the control and TO-SLIPS scalpel blades were tested against concentrated culture of *E. coli*. After sterilization, which did not compromise their wetting characteristics (Supplementary Fig. 11), the blades were submerged in a solution of liquid medium containing approximately 10^8^ cells per millilitre (Fig. 5f, insets). The results of the colony-forming units (c.f.u.) counts for the bacteria remaining on the blades are shown in Fig. 5f. The TO-SLIPS scalpel blades yielded a significantly lower remaining c.f.u. count compared with the control (*P*=0.025). Previous work examining bacteria in contact with SLIPS, made from Teflon membranes, has shown that the presence of the immobilized liquid layer can prevent bacterial biofilm formation in several species, even after 7 days of incubation in a concentrated solution of the organism^67^. This was attributed to the inability of the bacteria to attach to the lubricant surface, as any toxic effects of the lubricant on the bacteria have been ruled out^26^.

Another important application of steel is in the marine environment, where corrosion resistance to seawater and mechanical durability are critical, but marine fouling induces corrosion and drag, reduces speeds and significantly increases fuel costs^8^. Here we used the green alga *Chlamydomonas reinhardtii* as a model organism for aquatic fouling to explore the growth and adhesion of algal biofilms on TO-coated austenitic AISI 304 grade stainless-steel substrates. Untreated control, TO-SHS and TO-SLIPS stainless-steel samples were immersed in growth media with *C. reinhardtii* for 8 days. Over this growth period, there was a notable increase of algae density with no sign of substrate-related growth reduction or mortality, validating the non-toxic nature of the TO, TO-SHS and TO-SLIPS surfaces.

**Figure 6:**
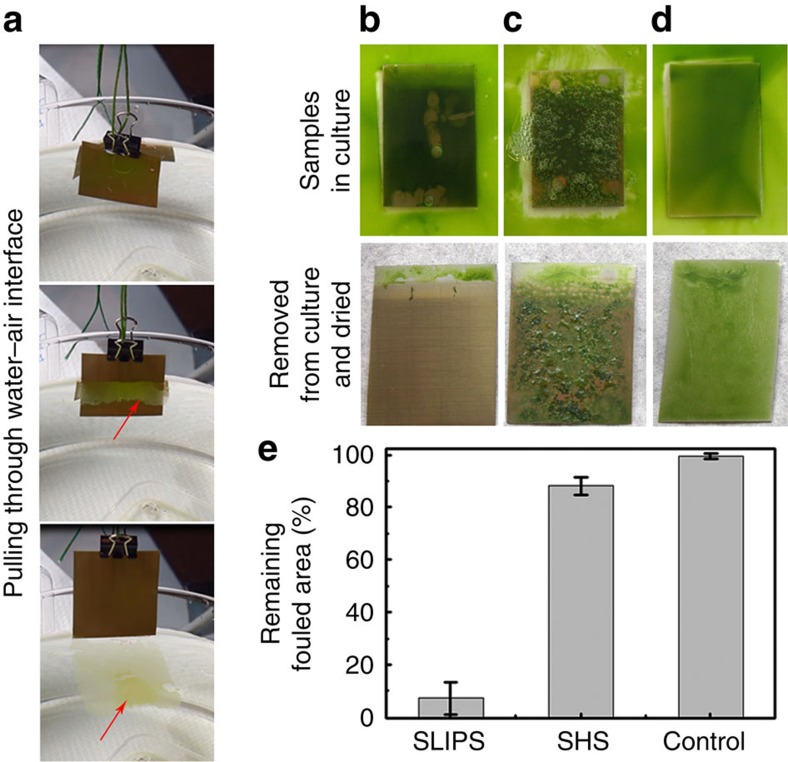
Resistance of TO-coated stainless steel to green algae biofouling. (**a**) Pulling of the TO-SLIPS sample from the media through the air–water interface after being exposed to *C. reinhardtii* culture for 8 days to grow biofilm. Images are still frames taken from Supplementary Movie 5. (**b**–**d**) The samples just before (top panels) and after (bottom panels) being removed from the culture and dried: (**b**) TO-SLIPS, (**c**) TO-SHS and (**d**) untreated stainless-steel control. (**e**) Area remaining fouled after the removal of the sample from the growth media followed by drying.

When the TO-SLIPS samples were pulled through the air–water interface (Fig. 6a), 93±6% of the attached biofilm was spontaneously delaminated leaving behind a largely clean surface with the exception of a few fragments attached to the upper untreated stainless-steel edge (Fig. 6b,e). The delamination of an interconnected, intact algal biofilm indicates that it is poorly adhered to the lubricant-coated underlying TO-SLIPS substrate allowing easy shedding of the attached algae during air–water interface transition. In contrast, biofilm was firmly attached to both TO-SHS and untreated steel surfaces during this removal process, leaving 88±4% and 99±1% fouled surfaces, respectively (Fig. 6c–e).

This performance difference between the TO-SHS and TO-SLIPS can be explained by the instability of the air layer trapped in the superhydrophobic surface under submerged conditions, especially when in contact with biological organisms, as compared to the stability of the lubricant layer in the slippery surface under the same conditions. Bacteria, for example, have been shown to use their flagella to displace the trapped air layer from the SHS, providing a new surface for attachment^68^. The exact mechanism by which the shedding of the biofilm occurs from the TO-SLIPS is still under investigation; however, a current hypothesis is that the algae form a film atop the lubricant overlayer, and therefore, have weak adhesion to the underlying solid surface. When transitioning through the air–water interface, the interfacial tension between the lubricant and water is sufficient to cause delamination of the biofilm^26^.

## Discussion

An electrochemical approach to deposit mechanically durable, hierarchically structured TO coating composed of randomly oriented nanoscale flakes on sub-microscale islands has been developed. TO coatings were deposited on steel from aqueous solution of sodium tungstate by pulsed electrochemical deposition at room temperature without use of any post-deposition treatments. Steels of various grades, including austenitic, ferritic, surgical stainless and naval ship building were modified. The intrinsically superhydrophilic, as-deposited TO coatings were converted to superhydrophobic by surface functionalization using fluorinated alkylphosphate ester. After fluorination of the structured surface, omniphobic-slippery surfaces were formed by infiltration of a fluorinated lubricant.

Chemical composition, structure, wetting and mechanical properties of the deposited TO films were analysed in detail. Here we summarize the main advantages of the TO deposits. (1) A facile and environmentally friendly electrodeposition method produces thin mechanically durable TO films on steel electrodes of various shapes, such as thin sheets, plates, meshes, molds, small inner diameter tubes, needles and so on. Furthermore, the TO films can be deposited in arbitrary microscale patterns by masking specific areas to enable spatially patterned durable repellent coatings. (2) The TO films made using our method are chemically attached to the underlying substrate through metal–oxygen–tungsten bonds that form during initial deposition cycles; further deposition develops island-like nanostructures. A combination of a chemically bonded coating with island-like morphology allows for excellent resistance to compressive, tensile and texture stresses, demonstrating unmatched mechanical durability and resistance to impact with hard and heavy particles (Supplementary Movie 1). (3) Using convenient wet chemistry, the deposited hierarchical films were converted to robust superhydrophobic (TO-SHS) coatings with water CA of ∼170°. Our TO-SHS surfaces show no decrease in their wetting characteristics after impacted with tens of thousands of YSZ particles with an impinging energy of >10^−4^ J per particle, which is three orders of magnitude higher than previously used in the sand abrasion experiments in which most mechanically durable SHS were tested. (4) As-deposited TO films are amorphous and oxygen-saturated, making the TO-SHS surfaces insensitive to the ultraviolet irradiation, which is extremely important for the stability and long-lasting anti-wetting performance in outdoor applications. (5) The TO-SHS coatings maintain their water-repelling performance after exposure to extreme temperatures in the range between −196 and +200 °C. Exposure to temperatures >200 °C does not change the TO surface morphology, but partially decomposes the fluorinated layer, which can be effectively restored by re-functionalization in the same surface modification solution. Moreover, when the TO-SHS surfaces were contaminated, annealing of these samples for 1 h at 200 °C restores completely their non-wetting performance. (6) When converted to the omniphobic-slippery TO surfaces, TO-SLIPS repel simple high- and low-surface-tension liquids (Supplementary Movie 2), complex fluids such as blood (Supplementary Movie 4), and significantly reduce adhesion of biological species such as bacteria and algae without toxic effects (Supplementary Movie 5). (7) The TO-SLIPS coatings retain their repellent properties and self-heal due to continuous self-lubrication even when surface is significantly damaged with highly abrasive materials, such as stainless-steel tweezers, a screw driver and a diamond scriber (Supplementary Movie 3) or after undergoing an intimate contact with or incision through soft, sticky materials, such as silicone elastomers or soft tissue.

These results suggest that omniphobic TO-SLIPS surfaces provide unmatched combination of extreme mechanical durability and anti-fouling properties, which could be particularly useful in applications that require mechanical operation under harsh environmental and fouling conditions. As described in the paper, one important area where such coatings can offer superior advantages is in the medical field where metals are broadly used as implants, surgical instruments, needles, to name a few. A mechanically durable, modified implant stainless-steel surface that prevents biofilm formation, without cytotoxic effects or risk of resistance development, would provide the desired protection against periprosthetic or device-associated infections that remain as a common and serious consequence of using indwelling implants in medical practice^13^. Surgical-grade stainless-steel instruments and devices with wear-resistive coating that sustain soft tissue incisions, improve incision edge smoothness, reduce shear and stickiness-related inaccuracies and, simultaneously, impede biofouling adhesion and blood clotting will be beneficial in prevention of the surgical site infections in either conventional or minimally invasive laparoscopic surgeries.

We also expect that our mechanically durable, temperature-stable omniphobic TO-SLIPS surfaces will find potential use in flow channels carrying viscous fluids. In particular, three-dimensional printing is emerging as one of the most promising fabrication technologies; however, since high pressure and heating are applied to melt and flow viscous inks, such as PDMS, polymeric gels or thick particle suspensions, through a sub-millimeter-scale steel nozzle, the process is highly energy consuming and surface contamination with the transported inks decreases the feature resolution and precision. Owing to semiconducting nature of the TO, the nozzles modified with TO-SHS and TO-SLIPS can be subjected to E-field-assisted droplet or jetting formation applications in which both conductivity and superhydrophobicity are required. On the larger scale, a mechanically durable TO-SLIPS coating that is stable under high temperature and pressure can be also applied in stainless-steel chemical reactors, to keep lubricant overlayer on internal reactor parts. It will prevent their clogging by the synthetic products and, therefore, will decrease energy losses and emission and increase the maintenance service period.

And finally, last but not least, we have demonstrated that TO-SLIPS can inhibit the attachment of aquatic microorganisms preventing bio-corrosion and fouling of steels broadly used in marine construction and maritime vessels, highlighting the possibility to apply these substrates as a novel type of non-toxic fouling-release coatings^23^. However, it is important to further investigate the seawater corrosion resistance according to seawater quality, temperature and oxygen content, and stability of the TO-SLIPS under dynamic flow conditions and field studies, in the case it is considered as a fouling-release coating in marine environment. While recent studies reported (a) the high stability of the lubricant layer in SLIPS constructed on aluminium modified with Al(O)(OH) nanostructures and subjected to high acceleration^52^, (b) retention of the lubricant on typical SLIPS surfaces in the flow-cell experiments performed at various flow rates^69^ and (c) biocompatibility of perfluorinated oils used as SLIPS lubricants^64^, a separate study specific to the performance of TO-SLIPS under various liquid flow conditions, its dependence on the viscosity and composition of the lubricant, feature size and shape of the tungsten oxide film as a function of the steel type and composition, as well as its biocompatibility, will be necessary.

## Methods

### Materials

Sodium tungstate dihydrate was purchased from Sigma-Aldrich (USA); MilliQ deionized (DI) water was used in all experiments; acetone and ethanol (200-Proof) were purchased from VWR. Phosphate ester with mixed length of fluorinated alkyl chains (FS-100) was purchased from Chemguard (USA). Krytox GPL-K103 lubricant was purchased from DuPont (USA). AISI 304, AISI 316 and AISI 430 stainless-steel plates, foils, rods and pipes were purchased from McMaster-Carr. Disposable stainless-steel surgical scalpels (Bard-Parker, USA) were purchased from VWR and HY-100 steel plates were received from Diversified Metals Inc., (USA). Zirconia (YSZ) grinding media was purchased from Inframat Advanced Materials Inc. (USA), and PDMS (Sylgard 184) was purchased from Dow Corning (USA).

### Surface fabrication

The electrochemical deposition process was performed in a standard three-electrode system using potentiostat/galvanostat (Princeton Applied Research, VersaSTAT3–200) and VersaStudio software (Princeton Applied Research). All measurements were carried out at room temperature without stirring or de-aeration of the solution. An aqueous solution of sodium tungstate (0.5 M) was used as the electrolyte (18.2 MΩ cm, Millipore Co., USA). Stainless-steel plates of various thicknesses (25 μm–0.5 cm) and grades (austenitic 304 and 316, ferritic 430, surgical and naval construction HY-100) or syringe needles and surgical scalpels were used as the working electrodes after cleaning with aqueous detergent (Alcojet, USA), acetone and deionized water and dried with N_2_ gas. The anode was a Pt gauze (10 × 20 mm) and the reference was an Ag|AgCl NaCl_(sat)_ electrode (BASi, MF-2052). All potential values are referred to this reference electrode throughout the paper. The cathodic electrochemical deposition was performed at a square-waveform pulse potential with pulse duration of 10 s separated by 10 s intervals. The typical potential of −1.5 V was applied for overall time of 12 h. After deposition, samples were removed from the solution, extensively rinsed with DI water and dried under nitrogen. TO-deposited samples were immersed in 1 w% fluorosurfactant (FS-100) dissolved in a mixture of 95:5 v/v% ethanol/water for 30 min at 70 °C or, alternatively, for 12 h at room temperature. After functionalization, samples were extensively rinsed with ethanol and dried under nitrogen. SLIPS samples were prepared by placing ∼10 μl cm^−2^ DuPont Krytox GPL-K103 on the modified surfaces, and uniform coverage was achieved by tilting. Excess lubricant was removed by vertical placement of the substrate or by spinning.

### Chemical characterization

A Zeiss FE-SEM Supra SEM equipped with an energy-dispersive X-ray analyser was used to determine the chemical composition of the porous TO films. A Zeiss FE-SEM Ultra Plus SEM with an In-lens secondary electron and Everhart–Thornley secondary electron detectors at acceleration voltage of 15 kV was used to determine the surface morphology. Surface composition was analysed using an X-ray photoelectron spectroscopy (XPS, K-Alpha Thermo Scientific) system with Al K-α X-rays. Core-level lines were calibrated to the C 1s peak (284.6 eV).

Raman measurements were performed using a Bruker Senterra Raman spectrometer equipped with a microscope (Bruker Optics, Billerica, MA). Measurements were carried out using 20 mW 532 nm laser focused through a × 100 objective. A spectral resolution of 3–5 cm^−1^ was used. The total exposure time was 2 × 30 s, which was found to be sufficient to obtain spectra with good signal-to-noise ratio without causing visible sample damage.

FTIR measurements were performed using a Bruker Hyperion 3000 FTIR Microscope/Vertex 70 FTIR Spectrometer equipped with a microscope (Bruker Optics, Billerica, MA) and the attenuated total reflectance objective with a germanium crystal. Measurements were carried out with a spectral resolution of 4 cm^−1^, 64 background and sample scans in the range of 600–4,000 cm^−1^.

### Mechanical characterization

Mechanical properties of stainless-steel substrates with and without deposited TO films were studied by nanoindentation technique (NanoIndenter G200, Agilent Technologies, USA). Continuous stiffness measurements mode was used to determine elastic modulus and hardness of the TO-deposited porous films. The nanoindenter was equipped with a Berkovich three-sided diamond pyramid indenter with centerline-to-face angle of 65.3° and a 17.8 nm radius at the tip of the indenter. The nanoindentation was carried out using a constant indentation strain rate of 0.05 s^−1^, continuous stiffness measurements amplitude was 2 nm with a frequency of 75 Hz and indent depth of 300 nm. The device is equipped with an optical microscope. Series of 15–25 indents were made for each sample probe, to obtain a better statistics as well as to cover large surface area. Distance between every measured point was 100 μm.

### Wetting characterization

Water CA, CAH and sliding angle measurements were performed using drop shape analysis system DSA100 (Kruss, Germany). Small droplets of water (5–10 μl) were placed on multiple areas over the surface of the samples and observed using a video camera. The angle was then estimated from the photos taken by the video camera using photo analysis software. The average CA, CAH and sliding angle were obtained by measuring at least five different locations on the sample.

### Biofouling experiments

Porcine whole blood in sodium heparin was purchased from Lampire Biological Laboratories (Pipersville, PA, catalogue no.: 7204911). The blood was used within 5 days of receipt. It was brought to room-temperature rotating on a mixer at 20 r.p.m. to homogenize the contents before sampling. Heparin was added to the blood before receipt (at the lab facility) as a way to prevent blood clotting. Tests against bacteria adhesion were performed by dipping a treated or untreated scalpel blade into a stock of *E. coli* strain J96 (∼108 cells per millilitre) in M63 medium. This clinically isolated *E. coli* strain was chosen owing to the role of *E. coli* as an infection-causing agent in many healthcare settings. The blades were then dipped in deionized, distilled water to remove excess bacteria. C.f.u. counts were performed by sonicating the blades in 1 × PBS for 10 min to remove the bacteria from the blades, then diluting and plating the resulting solution. There were four replicates per treatment. Statistical analysis (two-tailed *t*-test) was performed using SPSS Statistics 22 (IBM Corp., Armonk, NY).

The green alga *C. reinhardtii* (UTEX number 89) from the University of Texas Culture Collection was used as a model organism to explore the biofilm retention on TO surfaces. *C. reinhardtii* was grown in a Soil Extract (Bristol medium based) solution under non-axenic conditions until the stock culture reached a density of ∼1 × 10^7^ cells per millilitre. This stock culture was diluted with fresh Soil Extract to a 1:5 ratio of stock culture to fresh medium. The diluted culture was added to 80 ml square petri dishes, containing either lubricant-infused TO or control substrates, allowing the algae to settle on test surfaces. The petri dishes were then placed under a Sun Blaze T5HO fluorescent light fixture (Sunlight Supply, Inc., Vancouver, WA) and were grown under a 16:8 h light–dark cycle at 24 °C for 8 days. After 8 days, the samples were vertically lifted out of the culture medium at a controlled rate of 0.5 mm s^−1^ using a UMP3 syringe pump (World Precision Instruments Inc., Sarasota, Florida, USA). The treated substrates were immediately photographed after removal from culture to avoid subsequent biofilm retractions. The photos were then analysed using the Photoshop and ImageJ packages to assess total remaining biofilm coverage after air–water interface transition.

## Additional information

**How to cite this article:** Tesler, A. B. *et al.* Extremely durable biofouling-resistant metallic surfaces based on electrodeposited nanoporous tungstite films on steel. *Nat. Commun.* 6:8649 doi: 10.1038/ncomms9649 (2015).

## Supplementary Material

Supplementary Information Supplementary Figures 1-11

Supplementary Movie 1 Particle abrasion experiment of electrodeposited tungsten oxide films on stainless steel and wetting behavior after 10 abrasion tests.

Supplementary Movie 2 Omniphobic slippery TO films formed on stainless steel repel water, ethanol and silicone oil simultaneously.

Supplementary Movie 3 Self-healing properties and uncompromised liquid repellency of an omniphobic slippery TO film electrodeposited on stainless steel, while scratched with stainless steel tweezers, a screw driver, and a diamond pen.

Supplementary Movie 4 Blood adhesion to untreated scalpel, superhydrophobic TO-SHS and TO-SLIPS samples prepared on surgical grade scalpel blades.

Supplementary Movie 5 Pulling the sample with algae biofilm through the water-air interface.

## Figures and Tables

**Table 1:** Water contact angle, contact angle hysteresis and sliding angle of superhydrophobic (TO-SHS) and omniphobic (TO-SLIPS) coatings.

**Surface**	**Contact angle (deg.)**	**Contact angle hysteresis (deg.)**	**Sliding angle (deg.)**
TO-SHS	169±4	45±15	>45
TO-SLIPS	124±1	1.4±0.5	3.0±0.3
