# Supplementary material for: Extremely durable biofouling-resistant metallic surfaces based on electrodeposited nanoporous tungstite films on steel
Source: Nat Commun. 2015 Oct 20;6:8649. doi: 10.1038/ncomms9649 (PMC4667690; doi:10.1038/ncomms9649)
Supplement: Supplementary Information — Supplementary Figures 1-11 [file ncomms9649-s1.pdf]

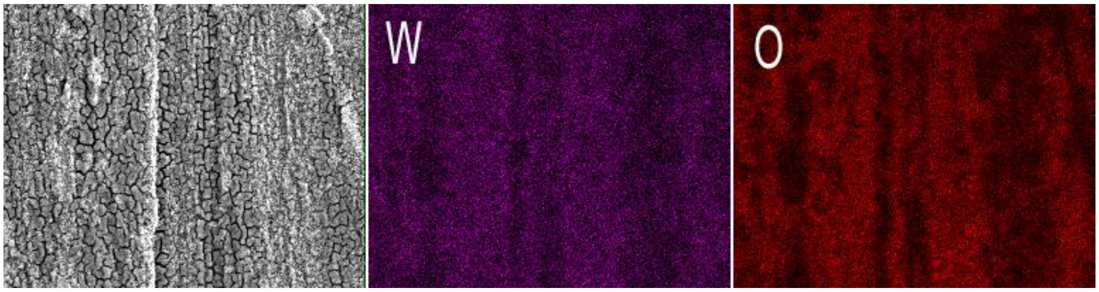

Supplementary Figure 1 | SEM image and corresponding EDAX maps of W and O on a 12 h pulsed electrodeposited tungsten oxide film on ASIS 304 grade stainless steel.

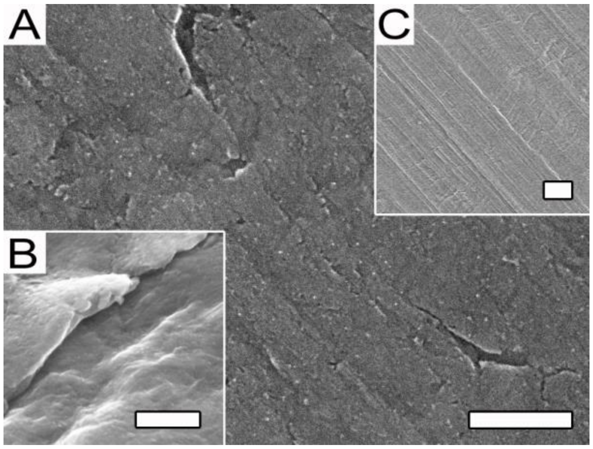

Supplementary Figure 2 | SEM images of tungsten oxide films formed by continuous electrochemical deposition on AISI 304 grade stainless steel. High magnification (**A**) top view and (**B**) tilted view (70°); (**C**) Low magnification top view. Scale bars are (A, B) 200 nm and (C) 10 µm.

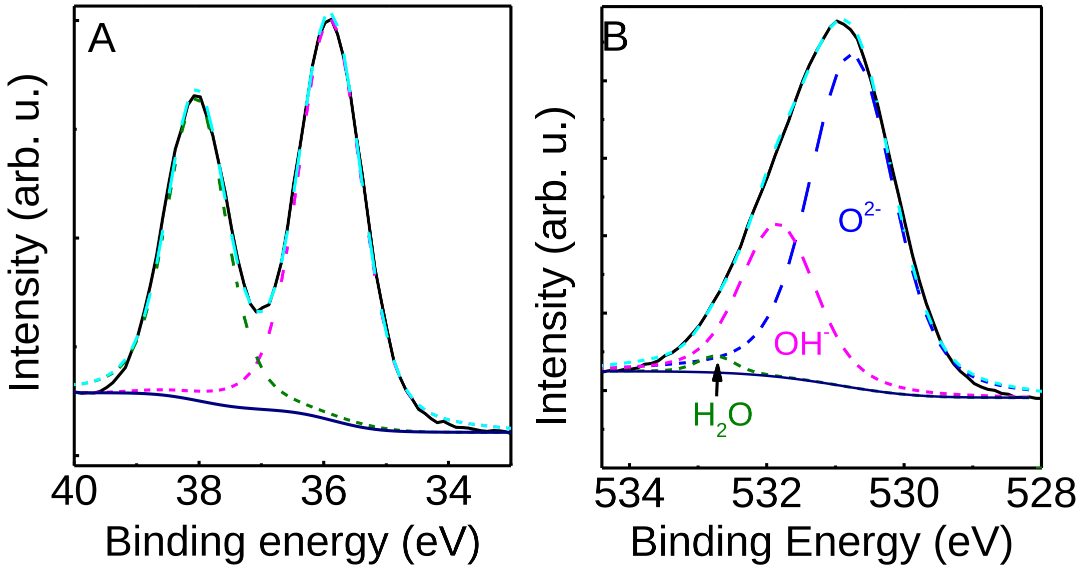

Supplementary Figure 3 | Measured and deconvoluted XPS spectra of (A) W 4f and (B) O 1s electrons of 12 h electrodeposited TO films on stainless steel substrates. Black line is measured spectrum and dashed line is deconvoluted calculated spectrum.

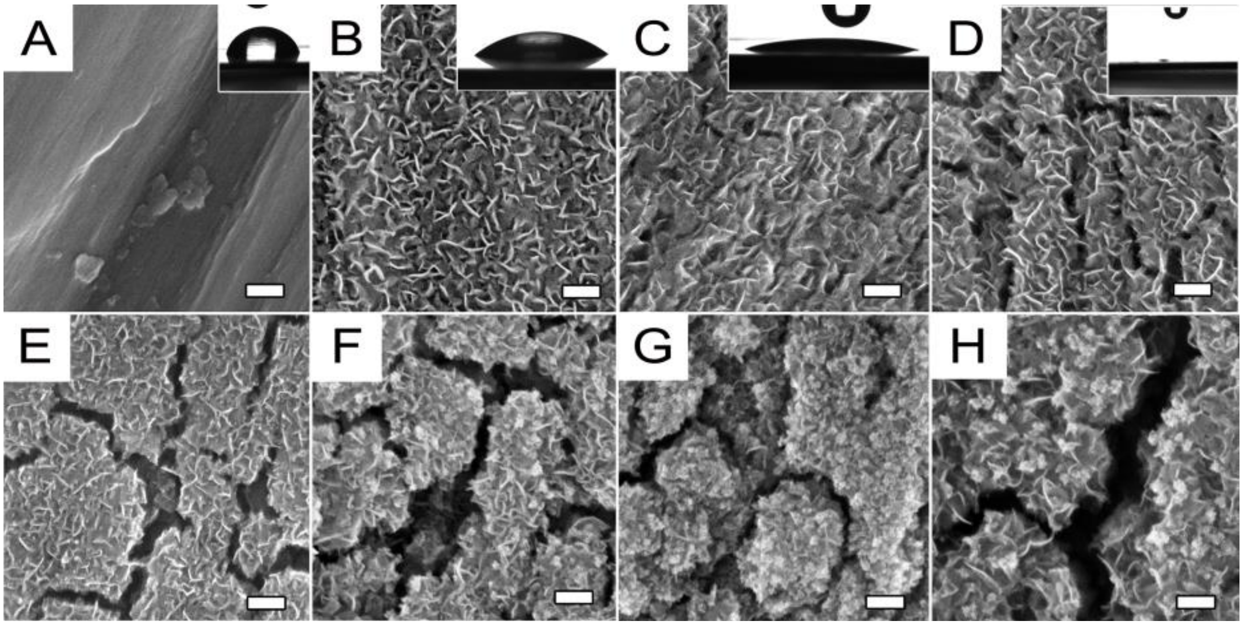

Supplementary Figure 4 | HR-SEM images showing the evolution of electrodeposited TO morphology on stainless steel as a function of electrodeposition time: (A) bare stainless steel, (B) 0.5 h, (C) 1 h, (D) 2 h, (E) 3 h, (F) 5 h, (G), 6 h and (H) 12 h. Inset pictures show the water contact angle on corresponding samples. Scale bars are 100 nm.

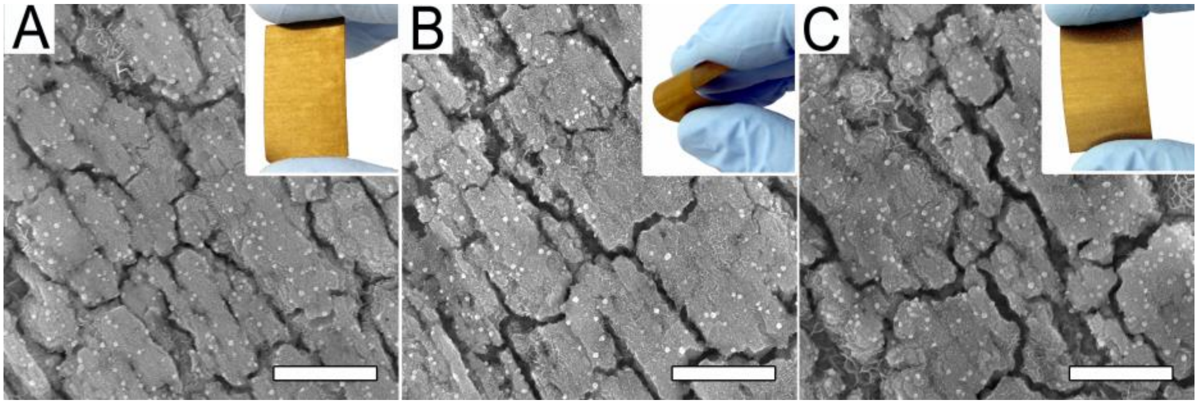

Supplementary Figure 5 | SEM images of electrochemically deposited TO films on a 100-μm-thick stainless steel foil before (**A**) and after (**C**) bending tests corresponding to Fig. 3B1 and 3B4, respectively. (**B**) Sample in the ~160° bended position as shown in Fig. 3B2. Scale bars are 1 μm.

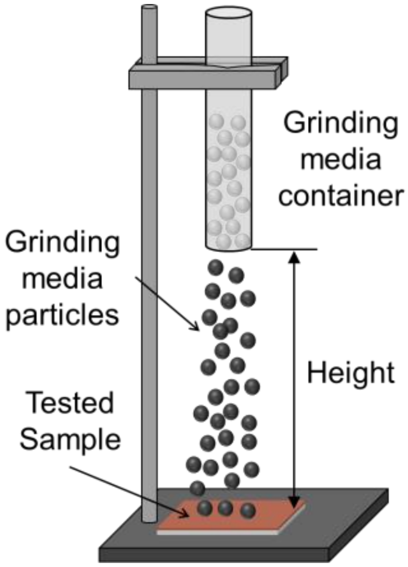

Supplementary Figure 6 | Schematic representation of a particle abrasion experiment setup. Surfaces were impinged with Yttria Stabilized Zirconia particles of 0.8 mm diameter (2.1 mg) and 2 mm diameter (28 mg) from a height of 40 cm over ~ 2 × 2 cm^2 area. 35 g of grinding media particles were used in every experiment corresponding to 1.6 × 10^5 particles of 0.8 mm and 1.2 × 10^3 particles of 2 mm diameter.

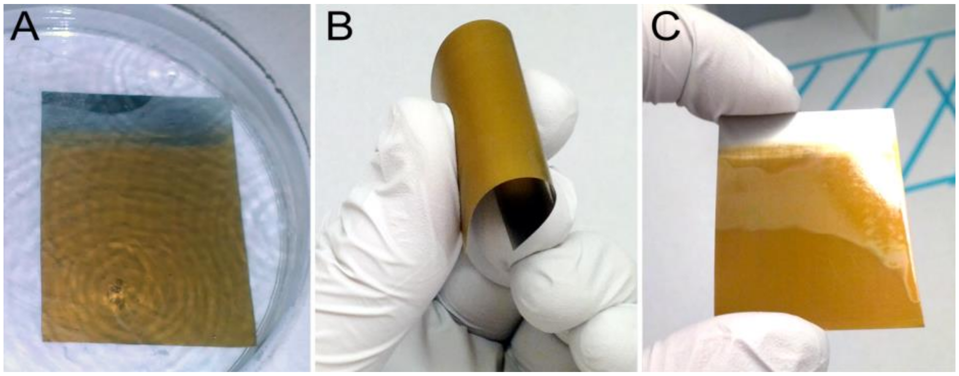

Supplementary Figure 7 | (**A**) As-prepared superhydrophilic TO films electrodeposited on a 50-µm-thick 304 grade stainless steel film were cooled at liquid nitrogen for few minutes. (**B**) Immediately after removing from the liquid nitrogen, samples were bent almost 180° a number of times. (**C**) Sample after bending test with residues of condensed water on superhydrophilic surface.

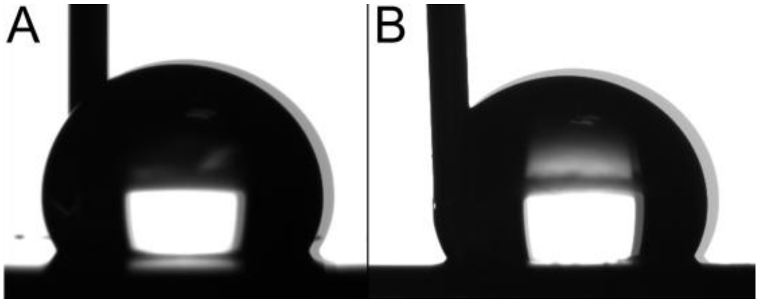

Supplementary Figure 8 | CAH images of a droplet on a sample measured just after lubrication (**A**) and a year after lubrication (**B**).

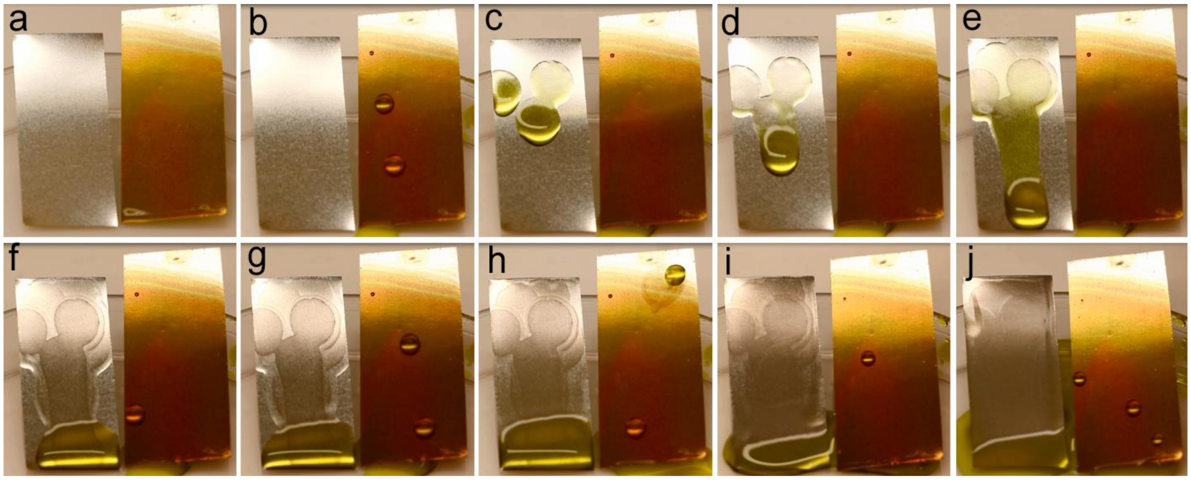

Supplementary Figure 9 | Accelerated corrosion test, in which unmodified stainless steel (300 grade) (left sample) and TO-SLIPS sample with a 600-nm-thick porous TO film on steel (right sample) were exposed to Ralph's etchant that contained 100 ml of H_2O, 200 ml of methyl alcohol, 100 ml of concentrated HCl, 2 gr $CuCl_2$, 7 gr $FeCl_2$, 5 ml of concentrated HNO_3. (**a-j**) Images show evolution of corrosion as a function of contact time. Mild corrosion is observed on the bare steel sample, and no change in TO-SLIPS sample.

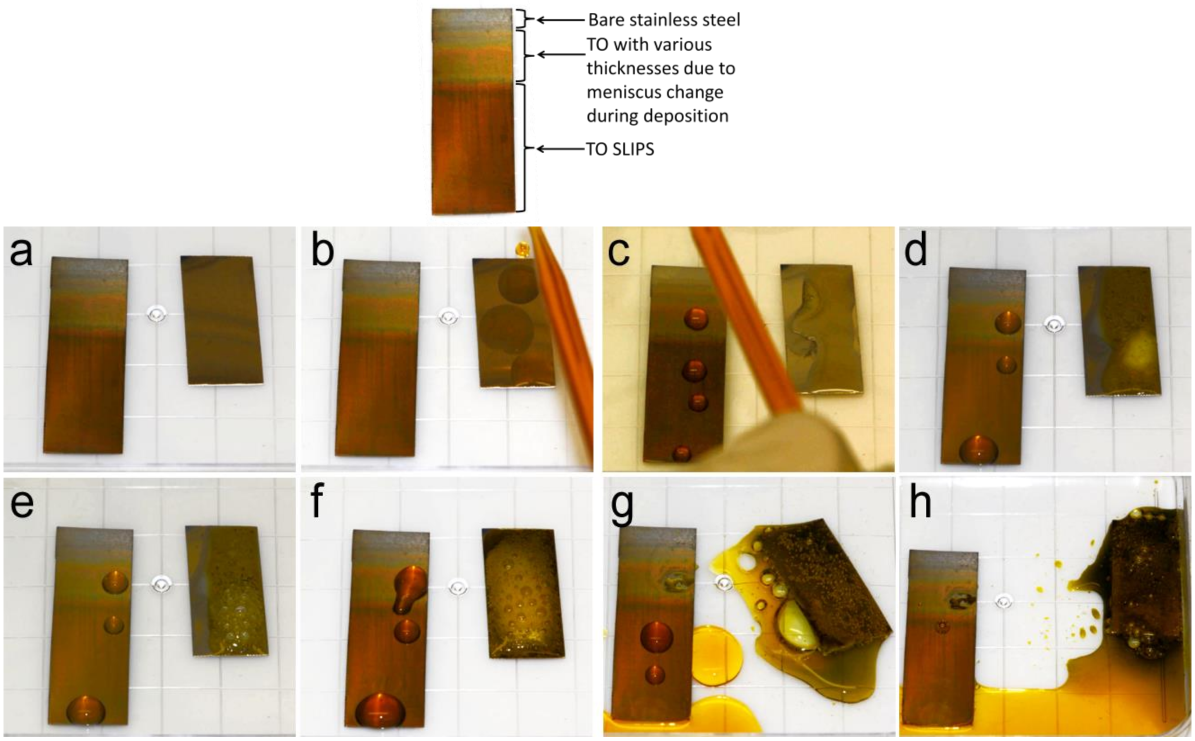

Supplementary Figure 10 | Accelerated corrosion test, in which unmodified stainless steel (300 grade) (right sample) and the lower part of the TO-SLIPS sample (shown as an inset on top) with a 600-nm-thick porous TO film on steel (left sample) were exposed to Glyceregia etchant that contained (according to ASTM E 407): 15 ml of concentrated HCl + 10 ml Glycerol + 5 ml of concentrated HNO_3. (**a-h**) Images show corrosion evolution as a function of contact time. Even

under such harsh chemical corrosion conditions, TO-SLIPS showed excellent corrosion resistance, while bare stainless steel sample corroded immediately after contact with the etchant that can be seen by a color change and formation of bubbles. When Glyceregia etchant was used on horizontally aligned samples, bare stainless steel sample was moving due to fast oxidation reaction.

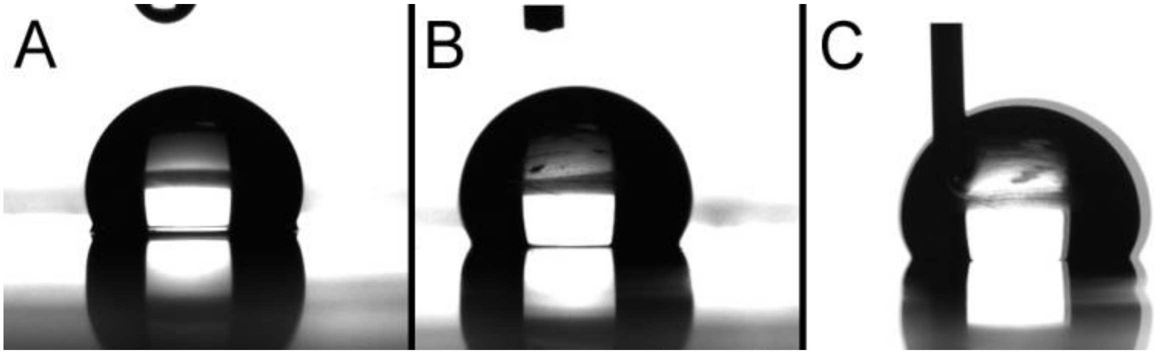

Supplementary Figure 11 | Water CA and CAH before and after sterilization of TO-SLIPS scalpels. The blades were first sterilized in either an ethanol/water mixture or autoclaved for 15 min at 121 ºC and then used in *E. coli* culture experiments. (**A**) Water CA on a surgical scalpel before sterilization; (**B**) water CA after sterilization and (**C**) CAH after autoclave sterilization.
